# Association of *PPP1R1B* polymorphisms with working memory in healthy Han Chinese adults

**DOI:** 10.3389/fnins.2022.989046

**Published:** 2022-11-10

**Authors:** Hui Ma, Riyang Qiu, Wenya Zhang, Xiaohong Chen, Liguo Zhang, Man Wang

**Affiliations:** ^1^Hainan Provincial Institute of Mental Health, Hainan Provincial Anning Hospital, Haikou, Hainan, China; ^2^Department of Precision Therapy, Shenzhen Kangning Hospital, Shenzhen, Guangdong, China; ^3^Department of Precision Therapy, Shenzhen Mental Health Center, Shenzhen, Guangdong, China; ^4^Department of Psychiatry, The Third Hospital of Heilongjiang Province, Bei’an, Heilongjiang, China

**Keywords:** *PPP1R1B*, gene polymorphisms, working memory, Wisconsin Card Sorting Test, Chinese

## Abstract

**Aims:**

The dopamine- and cAMP-regulated phosphoprotein (DARPP-32), which is encoded by the *PPP1R1B* gene, plays a converging regulatory role in the central nervous system by mediating the actions of dopamine, serotonin, and glutamate. Previous studies have demonstrated that variations in genes related to the dopamine system influence working memory. The present study thus investigated whether polymorphisms in *PPP1R1B* gene were associated with working memory.

**Materials and methods:**

A sample of 124 healthy Han Chinese were genotyped for three single nucleotide polymorphisms of *PPP1R1B* gene, namely rs12601930C/T, rs879606A/G, and rs3764352A/G, using polymerase chain reaction and restriction fragment length polymorphism analysis. Working memory performance was assessed using the Wisconsin Card Sorting Test (WCST).

**Results:**

Significant differences were observed in the Total Correct (TC), Total Errors (TE), and Conceptual Level Responses (CLR) scores of the WCST among the three rs12601930C/T genotypes (*p* = 0.044, 0.044, and 0.047, respectively); in TC, TE, Non-Perseverative Errors (NPE), and CLR scores between participants with the CC and (CT + TT) rs12601930C/T polymorphism genotypes (*p* = 0.032, 0.032, 0.019, and 0.029, respectively); in TC, TE, Perseverative Errors (PE), NPE, and CLR scores between participants with the (CT + CC) and TT rs12601930C/T polymorphism genotypes (*p* = 0.001, 0.001, 0.011, 0.004, and 0.001, respectively); and in NPE and CLR scores between participants with the GG and (AG + AA) genotypes of the rs3764352A/G polymorphism (*p* = 0.011 and 0.010). Furthermore, for males only, there were significant differences in TC, TE, PE, NPE, and CLR scores among the rs12601930C/T genotypes (*p* = 0.020, 0.020, 0.037, 0.029, and 0.014, respectively) and NPE and CLR scores among the rs3764352 genotypes (*p* = 0.045 and 0.042).

**Conclusion:**

*PPP1R1B* gene polymorphisms rs12601930C/T and rs3764352A/G might be associated with working memory assessed by the WCST in healthy Chinese adults, especially among males.

## Introduction

For decades, mental disorders have been classified based on their observed symptoms and disease course. However, it is still disputed to what extent disorders are distinct entities with boundaries. There is abundant evidence of genetic overlap of single nucleotide polymorphisms (SNPs) between multiple mental disorders, including schizophrenia (SCZ), bipolar disorder (BD), major depressive disorder (MDD), attention-deficit/hyperactivity disorder (ADHD), and autism spectrum disorder (ASD) (Cross-Disorder Group of the Psychiatric Genomics Consortoium, 2013; Cross-Disorder Group of the Psychiatric Genomics Consortoium et al., 2013; Brainstorm et al., 2018; Schork et al., 2019; Hammerschlag et al., 2020). Much attention has been paid to the evidence and feasibility of cross-disease diagnosis of mental disorders. Improved classification of mental disorders based on neurobiological measures requires a set of traits that map to transdiagnostic subgroups of patients and align with heritable, core psychopathological processes at the center of the disorders of interest (Schwarz et al., 2016). One promising candidate for this approach is working memory, for which deficits have been reported across multiple diagnostic entities including SCZ (Saykin et al., 1994; Gold et al., 1997; Schwarz et al., 2016; Zakic Milas and Milas, 2019; Ruiz-Sanchez et al., 2021), BD (Arts et al., 2008; Zakic Milas and Milas, 2019), MDD (Matsuo et al., 2007; Vance and Winther, 2021), ADHD (Ramos et al., 2020), and ASD (Rabiee et al., 2020).

Working memory refers to the ability to hold information “online” over time in order to perform a task; this kind of memory is encoded in the brain by persistent neural activity that outlasts the presentation of a stimulus (Bolton and Constantine-Paton, 2018). The working memory system, which maintains a limited set of representations for immediate use in cognition, is a central part of human cognition. Working memory is centrally involved in reasoning (Jolly et al., 2020), mindfulness (Li et al., 2021; Youngs et al., 2021), fluid intelligence (Brydges et al., 2021), attention (Vaughan and Laborde, 2021), language and vocabulary acquisition (Verhagen and Leseman, 2016), and a variety of other neurocognitive tasks (Ricker et al., 2018). The Wisconsin Card Sorting Test (WCST) is a complex, multifactorial test (Berg, 1948; Grant and Berg, 1948) that has been traditionally used to test frontal lobe function (Demakis, 2003; Liozidou et al., 2012). The WCST is a measure of many different neuropsychological functions including working memory, executive function, set-shifting capacity, and other cognitive processes (Gold et al., 1997; Rabin et al., 2005; Thurston-Snoha and Lewine, 2007; Liozidou et al., 2012; Lange et al., 2016; Kopp et al., 2021; Sogut et al., 2021). Successful WCST performance requires participants to remember their prior response and associated feedback and then use this information to select a new response. Although the ability to hold information in mind does not guarantee that a correct choice will be made, being unable to do so would preclude successful performance. Working memory is thus a necessary and important condition for successful WCST performance (Gold et al., 1997). Though the WCST is the gold standard for neuropsychological assessment of executive function (Rabin et al., 2005; Kopp et al., 2021), working memory and executive function overlap and interact with each other (Baddeley, 2012; Cristofori et al., 2019). For these reasons, the WSCT is used to assess working memory function in the present study.

At the molecular level, the neurotransmitter dopamine (DA) is a key regulatory component of executive function in the prefrontal cortex (PFC). Notably, dysfunction in dopaminergic circuitry can result in impaired working memory (Klaus and Pennington, 2019). Two established means of improving working memory performance, namely pharmacological and behavioral influences, are associated with similar biological mechanisms in the brain involving the dopaminergic system (Soderqvist et al., 2012). Working memory is a highly heritable cognitive trait with heritability estimates of up to 49% (Ando et al., 2001). Numerous studies have identified many common genetic variants that impact the function of the DA system could alter working memory performance (Soderqvist et al., 2012).

In 1983, the DA- and cyclic adenosine monophosphate (cAMP)-regulated phosphoprotein (DARPP-32) was first identified as a mediator in striatal neurons that receive dopaminergic neuron innervations (Walaas et al., 1983). DARPP-32, which is encoded by the *PPP1R1B* gene (located on 17q12), exhibits remarkable regional distribution in the brain that is roughly similar to that of DA innervations (Ouimet et al., 1984, 1992, 1998). Moreover, several studies have demonstrated that DARPP-32 plays a pivotal role in integrating signal transduction in dopaminoceptive neurons (Greengard et al., 1999; Svenningsson et al., 2004; Lin et al., 2021). When phosphorylated by protein kinase A (PKA) at threonine residue 34 (Thr34), DARPP-32 is converted into a potent inhibitor of protein phosphatase 1 (PP1); in contrast, when phosphorylated by cyclin-dependent kinase 5 (CDK5), it inhibits PKA (Svenningsson et al., 2004; Girault and Nairn, 2021). PP1 regulates the phosphorylation state and physiological activity of many neuronal phosphoproteins, including various neurotransmitter receptors, ion pumps, ion channels, and transcription factors (Svenningsson et al., 2004; Girault and Nairn, 2021). The effect of DARPP-32 is terminated by dephosphorylation at Thr34 by protein phosphatase 2B (PP2B, calcineurin) (Hernandez-Lopez et al., 2000). Thus, DARPP-32 is considered to play a converging regulatory role in the central nervous system by mediating the action of DA and various neurotransmitters acting on dopaminoceptive neurons, including serotonin and glutamate (Ouimet et al., 1998; Svenningsson et al., 2005).

Given the above evidence, the DARPP-32 system may relate to working memory function. However, there is no prior research on the association between *PPP1R1B* polymorphisms and working memory in the general Chinese population. To address this research gap, the present study investigated whether *PPP1R1B* variants were associated with performance on working memory, as assessed by WCST, in a healthy Chinese sample. Three *PPPP1R1B* SNPs, namely rs3764352A/G, rs879606A/G, and rs12601930C/T, were selected based on our previous studies of alcohol dependence (Ma et al., 2015), personality traits (Li et al., 2011b), defense mechanisms (Huang et al., 2013), and anxiety level (Ma et al., 2017), and other teams’ related studies of emotional leaning (Curcic-Blake et al., 2012) and cognitive performance (Kunii et al., 2014).

## Materials and methods

### Subjects

A total of 124 (55 males, 44.35%; 69 females, 55.65%) healthy undergraduate and graduate students were recruited from China Medical University, Shenyang, Liaoning Province, China. All participants were unrelated and of Han Chinese ethnic background. Individuals with a history of psychiatric, neurological, or severe/chronic physical illnesses were excluded. The age range was 20–25 years with a mean age ± standard deviation (SD) of 22.97 ± 1.55 years. The years of education ranged from 14 to 22 years (mean ± SD, 17.10 ± 1.76 years). There were no significant differences in age (*t* = 0.607, *p* = 0.545) or years of education (*t* = 1.374, *p* = 0.172) between males and females. All protocols in this study were approved by the Ethics Committee of China Medical University. Written informed consent was obtained from all participants.

### Neurocognitive assessment

The WCST was developed by Berg (1948) and Grant and Berg (1948), and its reliability in clinical practice falls into the desirable range (≥0.90) (Kopp et al., 2021). For this study, we used a simplified version of the WCST using 64 cards (WCST-64), which uses only half of the master cards but has similar validity (Axelrod, 2002). As the test time is shortened to 10–15 min, the WCST-64 is especially suitable for field operations and recording. We analyzed six sub-scales of the WCST: The numbers of Total Correct (TC), Total Errors (TE), Perseverative Responses (PR), Perseverative Errors (PE), and Non-Perseverative Errors (NPE), and Conceptual Level Responses (CLR). All participants were asked to complete the test alone within 20 min.

### Genotyping

A 2 ml sample of venous blood was obtained from each participant for genotyping. Amplification of gene fragments containing the *PPP1R1B* SNPs rs12601930C/T, rs879606A/G, and rs3764352A/G by polymerase chain reaction and subsequent genotyping by restriction fragment length polymorphism analysis were carried out as described in our previous studies (Li et al., 2011b; Huang et al., 2013; Ma et al., 2015, 2017).

### Statistical analysis

Data are presented as the percent frequency or mean and SD. Allele frequencies were calculated from the genotypes of each subject. The Hardy–Weinberg equilibrium (HWE) and genotype distributions of the three SNPs were assessed using the chi-square test. One-way analysis of variance (ANOVA) was used to compare the mean WCST scores between the three different genotypic groups. The independent sample *t-*test was used to compare the mean age, years of education, and WCST scores between male and female participants and WCST scores between the two dominant-model groups or two recessive-model groups. The level of statistical significance was set at *p* < 0.05. The Statistical Package for the Social Sciences (SPSS 22.0 for Windows, SPSS Inc., Chicago, IL, USA) was used to perform the above statistical analyses.

## Results

### Hardy–Weinberg equilibrium results

Table 1 shows the genotype counts and frequencies for the three studied polymorphisms. The genotype distributions of rs879606 A/G (χ^2^ = 1.620, *p* = 0.203) and rs3764352 A/G (χ^2^ = 1.233, *p* = 0.267) did not deviate significantly from HWE, while the genotype distribution of rs12601930C/T deviated from HWE (χ^2^ = 5.834, *p* = 0.016). The genotype frequency of rs12601930C/T in the present study was not significantly different from that observed in a previous sample of 82 Han Chinese individuals^1^ (χ^2^ = 2.187, *p* = 0.335). Therefore, the sample in the present study can be considered representative of the general Han Chinese population.

**TABLE 1:** Effects of *PPP1R1B* polymorphisms on WCST scores.

SNP	Genotype	*N*(%)	WCST					
			TC	TE	PR	PE	NPE	CLR
rs12601930	CC	70 (56.45%)	47.64 ± 9.02	16.36 ± 9.02	9.19 ± 6.09	8.44 ± 5.14	7.91 ± 5.26	42.89 ± 13.58
	CT	39 (31.45%)	49.67 ± 6.76	14.33 ± 6.76	8.72 ± 5.19	7.92 ± 4.16	6.41 ± 3.80	45.97 ± 9.33
	TT	15 (12.10%)	53.13 ± 4.09	10.87 ± 4.09	6.20 ± 3.41	5.80 ± 2.96	5.07 ± 2.28	50.80 ± 5.39
	*F-*value		3.209	3.209	1.783	2.006	3.034	3.128
	*P-*value		0.044	0.044	0.172	0.139	0.052	0.047
Dominant-model	CC	70 (56.45%)	47.64 ± 9.02	16.36 ± 9.02	9.19 ± 6.09	8.44 ± 5.14	7.91 ± 5.26	42.89 ± 13.58
	CT + TT	54 (43.55%)	50.63 ± 6.30	13.37 ± 6.30	8.02 ± 4.86	7.33 ± 3.96	6.04 ± 3.48	47.31 ± 8.65
	*t-*value		−2.169	2.169	1.154	1.314	2.385	−2.208
	*P-*value		0.032	0.032	0.251	0.191	0.019	0.029
Recessive-model	CT + CC	109 (87.90%)	48.37 ± 8.31	15.63 ± 8.31	9.02 ± 5.76	8.26 ± 4.80	7.38 ± 4.83	43.99 ± 12.28
	TT	15 (12.10%)	53.13 ± 4.09	10.87 ± 4.09	6.20 ± 3.41	5.80 ± 2.96	5.07 ± 2.28	50.80 ± 5.39
	*F-*value		−3.607	3.607	1.847	2.757	3.083	−3.738
	*P-*value		0.001	0.001	0.067	0.011	0.004	0.001
rs879606	AA	27 (21.77%)	51.30 ± 6.52	12.70 ± 6.52	7.93 ± 5.50	7.22 ± 4.40	5.48 ± 2.89	48.59 ± 8.66
	AG	54 (43.55%)	48.83 ± 7.40	15.17 ± 7.40	8.56 ± 4.91	7.94 ± 4.13	7.22 ± 4.56	44.37 ± 11.94
	GG	43 (34.68%)	47.60 ± 9.44	16.40 ± 9.44	9.30 ± 6.46	8.44 ± 5.48	7.95 ± 5.43	43.00 ± 13.14
	*F-*value		1.772	1.772	0.521	0.560	2.435	1.941
	*P-*value		0.174	0.174	0.595	0.572	0.092	0.148
Dominant-model	AA	27 (21.77%)	51.30 ± 6.52	12.70 ± 6.52	7.93 ± 5.50	7.22 ± 4.40	5.48 ± 2.89	48.59 ± 8.66
	AG + GG	97 (78.23%)	48.29 ± 8.35	15.71 ± 8.35	8.89 ± 5.63	8.16 ± 4.75	7.55 ± 4.95	43.76 ± 12.44
	*t-*value		1.729	−1.729	−0.788	−0.926	−1.986	1.891
	*P-*value		0.086	0.086	0.432	0.356	0.052	0.061
Recessive-model	AG + AA	81 (65.32%)	49.65 ± 7.18	14.35 ± 7.18	8.35 ± 5.09	7.70 ± 4.21	6.64 ± 4.15	45.78 ± 11.08
	GG	43 (34.68%)	47.60 ± 9.44	16.40 ± 9.44	9.30 ± 6.46	8.44 ± 5.48	7.95 ± 5.43	43.00 ± 13.14
	*t-*value		1.353	−1.353	−0.906	−0.835	−1.503	1.244
	*P-*value		0.179	0.179	0.367	0.405	0.136	0.216
rs3764352	AA	42 (33.87%)	47.57 ± 9.78	16.43 ± 9.78	8.93 ± 6.40	8.21 ± 5.53	8.21 ± 5.59	42.74 ± 13.77
	AG	55 (44.36%)	48.89 ± 7.14	15.11 ± 7.14	8.84 ± 5.17	8.09 ± 4.21	7.02 ± 4.35	44.38 ± 11.56
	GG	27 (21.77%)	51.19 ± 6.49	12.81 ± 6.49	7.96 ± 5.21	7.30 ± 4.22	5.52 ± 3.02	48.93 ± 7.98
	*F-*value		1.673	1.673	0.281	0.352	2.861	2.354
	*P-*value		0.192	0.192	0.755	0.704	0.061	0.099
Dominant-model	GG	27 (21.77%)	51.19 ± 6.49	12.81 ± 6.49	7.96 ± 5.21	7.30 ± 4.22	5.52 ± 3.02	48.93 ± 7.98
	AG + AA	97 (78.23%)	48.32 ± 8.37	15.68 ± 8.37	8.88 ± 5.71	8.14 ± 4.80	7.54 ± 4.93	43.67 ± 12.52
	*t-*value		1.898	−1.898	−0.749	−0.832	−2.631	2.636
	*P-*value		0.063	0.063	0.455	0.407	0.011	0.010
Recessive-model	AG + GG	82 (66.13%)	49.65 ± 6.98	14.35 ± 6.98	8.55 ± 5.17	7.83 ± 4.21	6.52 ± 4.00	45.88 ± 10.68
	AA	42 (33.87%)	47.57 ± 9.78	16.43 ± 9.78	8.93 ± 6.40	8.21 ± 5.53	8.21 ± 5.59	42.74 ± 13.77
	*t-*value		1.224	−1.224	−0.357	−0.432	−1.744	1.401
	*P-*value		0.225	0.225	0.722	0.666	0.086	0.164

Wisconsin Card Sorting Test data present the means ± standard deviation. The six WCST variables are Total Correct (TC), Total Errors (TE), Perseverative Responses (PR), Perseverative Errors (PE), Non-Perseverative Errors (NPE), and Conceptual Level Responses (CLR).

### Relationship between *PPP1R1B* polymorphisms and working memory in the overall sample

Table 1 presents the WCST scores. Significant differences were demonstrated in the TC, TE, and CLR scores among three rs12601930 genotypes (*F* = 3.209, 3.209, and 3.128; *p* = 0.044, 0.044, and 0.047, respectively). The order of the above scores for the different genotypes was as follows: CC < CT < TT for TC and CLR, and TT < CT < CC for TE for the rs12601930 polymorphism. No significant differences were observed for any WCST results among the rs879606 or rs3764352 genotypes (all *p* > 0.05).

Next, the three genotypes for each SNP were divided into two different groups based on recessive and dominant-models. As shown in Table 1, there were significant differences in the TC, TE, NPE, and CLR scores of WCST between participants with the CC and (CT + TT) genotypes of the rs12601930 polymorphism. Participants with the CC genotype had lower TC and CLR scores (*t* = −2.169 and −2.208; *p* = 0.032 and 0.029, respectively) and higher TE and NPE scores (*t* = 2.169 and 2.385; *p* = 0.032 and 0.019, respectively) than those with the (CT + TT) genotypes. There were also significant differences in the TC, TE, PE, NPE, and CLR scores between participants with the (CT + CC) and TT genotypes of the rs12601930 polymorphism. Participants with the (CT + CC) genotypes showed lower TC and CLR scores (*t* = −3.607 and −3.738, *p* = 0.001 and 0.001, respectively) and higher TE, PE, and NPE scores (*t* = 3.607, 2.757, and 3.083; *p* = 0.001, 0.011, and 0.004, respectively) than those with the TT genotype.

In addition, there were significant differences in NPE and CLR scores between participants with the GG and (AG + AA) genotypes of the rs3764352 polymorphism. Participants with the GG genotype showed lower NPE scores (*t* = −2.631, *p* = 0.011) and higher CLR scores (*t* = 2.636, *p* = 0.010) than those with the (AG + AA) genotypes. However, there was no significant difference for any variable of the WCST between the two models of rs879606 A/G genotypes (all *p* > 0.05).

### Relationship between *PPP1R1B* polymorphisms and working memory in male and female participants

Differences in WCST scores among the genotype groups of the three studied polymorphisms were analyzed separately by sex. As shown in Table 2, there were significant male-specific differences in TC, TE, PE, NPE, and CLR scores among the rs12601930 genotypes (*F* = 4.213, 4.213, 3.502, 3.810, and 4.637; *p* = 0.020, 0.020, 0.037, 0.029, and 0.014, respectively), as well as in NPE and CLR scores among the rs3764352 genotypes (*F* = 3.303 and 3.384; *p* = 0.045 and 0.042, respectively). There were no significant male-specific differences in any WCST measure among the rs879606 genotypes (all *p* > 0.05).

**TABLE 2:** Effects of *PPP1R1B* polymorphisms on WCST scores in males.

SNP	Genotype	*N*(%)	WCST					
			TC	TE	PR	PE	NPE	CLR
rs12601930	CC	27 (49.09%)	45.22 ± 10.83	18.78 ± 10.83	11.52 ± 7.50	10.41 ± 6.22	8.37 ± 5.39	38.78 ± 16.77
	CT	20 (36.36%)	49.85 ± 7.61	14.15 ± 7.61	8.80 ± 5.73	8.10 ± 4.76	6.05 ± 3.49	46.45 ± 9.81
	TT	8 (14.55%)	55.13 ± 1.81	8.88 ± 1.81	5.50 ± 2.07	5.00 ± 1.31	3.88 ± 1.13	53.88 ± 1.73
	*F-*value		4.213	4.213	3.032	3.502	3.810	4.637
	*P-*value		0.020	0.020	0.057	0.037	0.029	0.014
rs879606	AA	14 (25.46%)	52.36 ± 7.98	11.64 ± 7.98	7.00 ± 5.67	6.36 ± 4.72	5.29 ± 3.73	50.14 ± 9.49
	AG	26 (47.27%)	48.42 ± 8.48	15.58 ± 8.48	9.62 ± 5.63	8.81 ± 4.55	6.77 ± 4.51	43.27 ± 14.02
	GG	15 (27.27%)	44.47 ± 11.26	19.53 ± 11.26	12.20 ± 8.26	11.00 ± 6.97	8.53 ± 5.18	38.67 ± 16.35
	*F-*value		2.666	2.666	2.353	2.729	1.877	2.555
	*P-*value		0.079	0.079	0.105	0.075	0.163	0.087
rs3764352	AA	13 (23.64%)	43.46 ± 12.41	20.54 ± 12.41	11.85 ± 8.67	11.00 ± 7.46	9.54 ± 5.74	36.46 ± 18.05
	AG	26 (47.27%)	48.73 ± 8.06	15.27 ± 8.06	9.85 ± 5.86	8.88 ± 4.63	6.38 ± 4.07	43.81 ± 13.30
	GG	16 (29.09%)	51.69 ± 7.68	12.31 ± 7.68	7.56 ± 5.57	6.81 ± 4.58	5.50 ± 3.69	49.63 ± 9.01
	*F-*value		2.939	2.939	1.558	2.164	3.303	3.384
	*P-*value		0.062	0.062	0.220	0.125	0.045	0.042

The differences in WCST scores among the three *PPP1R1B* genotype groups in female participants are shown in Table 3. Notably, there was no significant female-specific difference in any variable for the three studied SNPs (all *p* > 0.05).

**TABLE 3:** Effects of *PPP1R1B* polymorphisms on WCST scores in females.

SNP	Genotype	*N*(%)	WCST					
			TC	TE	PR	PE	NPE	CLR
rs12601930	CC	43 (62.32%)	49.16 ± 7.40	14.84 ± 7.40	7.72 ± 4.51	7.21 ± 3.93	7.63 ± 5.22	45.47 ± 10.55
	CT	19 (27.54%)	49.47 ± 5.95	14.53 ± 5.95	8.63 ± 4.71	7.74 ± 3.56	6.79 ± 4.17	45.47 ± 9.05
	TT	7 (10.14%)	50.86 ± 4.88	13.14 ± 4.88	7.00 ± 4.55	6.71 ± 4.07	6.43 ± 2.57	47.29 ± 6.10
	*F-*value		0.186	0.186	0.413	0.216	0.327	0.108
	*P-*value		0.831	0.831	0.664	0.806	0.722	0.898
rs879606	AA	13 (18.84%)	50.15 ± 4.53	13.85 ± 4.53	8.92 ± 5.35	8.15 ± 4.00	5.69 ± 1.70	46.92 ± 7.69
	AG	28 (40.58%)	49.21 ± 6.38	14.79 ± 6.38	7.57 ± 3.99	7.14 ± 3.59	7.64 ± 4.65	45.39 ± 9.77
	GG	28 (40.58%)	49.29 ± 8.04	14.71 ± 8.04	7.75 ± 4.74	7.07 ± 3.99	7.64 ± 5.62	45.32 ± 10.68
	*F-*value		0.093	0.093	0.414	0.396	0.900	0.134
	*P-*value		0.911	0.911	0.663	0.675	0.411	0.874
rs3764352	AA	29 (42.03%)	49.41 ± 7.93	14.59 ± 7.93	7.62 ± 4.70	6.97 ± 3.96	7.62 ± 5.52	45.55 ± 10.56
	AG	29 (42.03%)	49.03 ± 6.34	14.97 ± 6.34	7.93 ± 4.37	7.38 ± 3.75	7.59 ± 4.58	44.90 ± 9.96
	GG	11 (15.94%)	50.45 ± 4.46	13.55 ± 4.46	8.55 ± 4.85	8.00 ± 3.74	5.55 ± 1.81	47.91 ± 6.47
	*F-*value		0.172	0.172	0.163	0.299	0.878	0.381
	*P-*value		0.842	0.842	0.850	0.742	0.420	0.685

### Comparison of *PPP1R1B* polymorphisms’ genotype distributions and working memory between male and female participants

No significant differences were found in the genotype distributions of the *PPP1R1B* SNPs rs6090453, rs6011914, and rs2427422 (χ^2^ = 2.197, 2.492, and 5.677, *p* = 0.333, 0.288, and 0.059, respectively) or in any of the six WCST scores between male and female participants (Table 4, all *p* > 0.05).

**TABLE 4:** Comparation of WCST scores between male and female participants.

WCST	variable scores (Mean ± SD)		*t*-value	*P-value*
	Male (*N* = 55)	Female (*N* = 69)		
TC	48.35 ± 9.48	49.42 ± 6.75	−0.710	0.480
TE	15.65 ± 9.48	14.58 ± 6.75	0.710	0.480
PR	9.65 ± 6.61	7.90 ± 4.53	1.751	0.082
PE	8.78 ± 5.52	7.30 ± 3.80	1.692	0.094
NPE	6.87 ± 4.60	7.28 ± 4.72	−0.478	0.634
CLR	43.76 ± 14.14	45.65 ± 9.70	−0.845	0.400

## Discussion

The present study used 124 unrelated healthy Han Chinese participants to investigate associations between variants of the *PPP1R1B* gene and working memory function assessed by the WCST for the first time. We found that the rs12601930 (three genotypes, dominant or recessive-models) and rs3764352 (dominant-model) polymorphisms were significantly associated with some dimensions of working memory in the total sample. Moreover, these associations were demonstrated to be specific to males. The dimensions of working memory showing statistically significant differences included TC, TE, PE, NPE, and CLR among the rs12601930 groups, and NPE and CLR among the rs3764352 groups.

Working memory, which is a core part of human cognition, is a limited capacity system that integrates and manipulates information over brief periods of time and engages a network of prefrontal, parietal, and subcortical regions (Duncan et al., 2000; Gray et al., 2003). In SCZ, deficits of working memory have been found on various neuropsychological tests (Saykin et al., 1994; Karlsgodt et al., 2011; Schwarz et al., 2016; Ruiz-Sanchez et al., 2021). Working memory impairments in SCZ occur independently of antipsychotic medication use and appear to represent trait-like, rather than state-like, impairments with high consistency across symptom fluctuations and with little correlation to symptom severity (Gur et al., 2007; Schwarz et al., 2016). Meta-analyses have demonstrated the role of working memory deficits in ADHD (Ramos et al., 2020) and BD (Arts et al., 2008), and a number of neuropsychological studies support working memory deficits in MDD patients (Matsuo et al., 2007; Vance and Winther, 2021). Therefore, working memory is a promising candidate for cross-disease diagnosis of psychiatric diseases. Schwarz et al. (2016) adopted working memory as intermediate phenotype for improved illness classification. Previous studies have demonstrated that genetic factors are an important basis for common and stable changes in working memory function across various mental disorders (Gur et al., 2007; Karlsgodt et al., 2011). Twin studies suggest that a substantial part (up to 43%) of the genetic variance related to working memory modalities is due to a common genetic factor, with additional genetic variance explained by modality-specific factors (Ando et al., 2001). However, the genetic underpinnings of working memory have not been yet fully uncovered.

At the molecular level, the neurotransmitter DA is a key regulatory component of executive function in the PFC, and dysfunction in dopaminergic circuitry has been shown to result in impaired working memory (Klaus and Pennington, 2019). Pharmacological studies suggest that DA and DA agonists in the brain modulate delayed response tasks and working memory (Kimberg and D’Esposito, 2003; Vijayraghavan et al., 2007). In a randomized, double-blind, placebo-controlled study, Furman et al. (2021) demonstrated that augmenting cortical DA tone preferentially improved working memory maintenance. Prior research has found that multiple common genetic variants that impact the DA system, including DA receptors (DR), catechol-O-methyltransferase (COMT), the dopamine transporter (DAT), etc., could also alter working memory task performance. Functional genetic variants influencing D2-receptor function (DRD2 rs1076560) and Akt1 abundance implicated in downstream D2-signal transduction (AKT1 rs1130233) have been repeatedly found to affect prefrontal blood oxygen level-dependent activity during working memory, consistent with the complex role of prefrontal DA in human working memory (Emamian et al., 2004; Beaulieu et al., 2005; Harris et al., 2005; Zhang et al., 2007; Tan et al., 2008; Bertolino et al., 2009; Giovannetti et al., 2010). Caldu et al. (2007) found an additive effect of the COMT Val108/158 Met polymorphism and the 9-repeat allele of the DAT 40 base pair variable number of a tandem repeat polymorphism on brain activation during an N-back task in healthy subjects. They also demonstrated that the Val allele was related to higher number of PE on the WCST and a higher number of commission errors on the Continuous Performance Test (CPT) (Caldu et al., 2007). Wilkosc et al. (2010) reported an association between *DRD1*, *DRD4*, and *COMT* polymorphisms and performance on the WCST in healthy volunteers. Dumontheil et al. (2014, 2020) demonstrated that variation in *COMT* was associated with performance on verbal and visuospatial working memory tasks in adults and that the pattern of better working memory performance in Met/Met individuals observed in adulthood emerges during development, which is consistent with decreased levels of prefrontal DA during adolescence. Moreover, Li et al. (2011a) reported that polymorphisms in the *NTR1* gene, which is also strongly linked to the DA system, were associated with working memory performance assessed with a 2-back working memory paradigm in healthy Chinese undergraduates. The above reports support the results of the present study since DARPP-32 is a key component of DA signaling.

The relationship between DARPP-32 and working memory has been reported at the protein level among patients with mental disorders. Ishikawa et al. (2007) examined the distribution and expression of DARPP-32 in the post-mortem dorsolateral prefrontal cortex (DLPFC) of 12 patients with SCZ, 10 patients with BD, and 11 controls, and found that DARPP-32 was decreased in the DLPFC of patients with SCZ and BD compared to controls. Kunii et al. (2019) examined the PFC and nucleus accumbens (NAc) of 49 post-mortem patients with SCZ, BD, and normal controls. They reported that DARPP-32 levels in the PFC of patients with SCZ were significantly decreased, while levels of DARPP-32 in the NAc showed no significant alternations in patients with SCZ or BD. Torres et al. (2009) measured DARPP-32 expression in blood cell sub-populations (CD4 + T lymphocytes, CD56 + NK cells, CD19 + B lymphocytes, and CD14 + monocytes) and found that DARPP-32 expression was diminished in CD4 + T lymphocytes, CD19 + B lymphocytes, and CD14 + monocytes of BPD patients and also decreased in CD4 + T lymphocytes and CD56 + NK cells of SCZ patients. Thus, at the protein level, DARPP-32 may underlie working memory, since deficits in working memory always occur in patients with SCZ or BD (Gold et al., 1997).

Direct studies of the relationship between *PPP1R1B* variants and working memory are rare. Curcic-Blake et al. (2012) demonstrated that homozygotes with GTA alleles of the three SNPs of *PPP1R1B* (rs879606A/G, rs907094T/C, and rs3764352A/G) might engage in a greater degree of motivational learning and integration of information to correctly perform an emotional learning task. Houlihan et al. (2009) suggested that the *PPP1R1B* gene merits further attention for association with cognitive ability and/or age-related cognitive change. Kunii et al. (2014) demonstrated that the increased expression of truncated-DARPP-32 in the DLPFC of patients with SCZ and BD was strongly associated with *PPP1R1B* genotypes at SNPs rs879606, rs90974, and rs3764352 in a sample of 709 post-mortem brains, and that *PPP1R1B* genetic variants predicting worse cognitive performance were associated with higher truncated-DARPP-32 expression. Another study found that a frequent 7-SNP *PPP1R1B* haplotype could predict mRNA expression of DARPP-32 isoforms in the post-mortem human brain and was associated with enhanced performance on several cognitive tests depending on frontostriatal function (Meyer-Lindenberg et al., 2007). Although two SNPs of *PPP1R1B* gene, namely rs879606 and rs3764352, were investigated in the above studies and the present study, the previous studies assessed general cognitive ability or learning ability and most subjects were post-mortem patients with mental disorders, which differs from the present study.

In the present study, we demonstrated no significant differences in the performances of all six dimensions of the WCST or genotype distributions of the three studied SNPs between males and females, indicating no sex-specific difference in working memory function or *PPP1R1B* genotype distribution. However, there were significant male-specific differences in TC, TE, PE, NPE, and CLR scores among the rs12601930 genotypes and NPE and CLR scores among the rs3764352 genotypes. Therefore, the interaction of *PPP1R1B* polymorphisms and sex could influence individual working memory. Previous studies have shown that the interaction of DA-related polymorphisms and sex can influence working memory (Wilkosc et al., 2010; Wu et al., 2020). One potential reason for sex-specific findings is androgens, which might be involved in the effect of *PPP1R1B* on DA and other neurotransmitter systems, and thus ultimately affect the biological determination of working memory. Biochemical experiments will be needed to test if this conjecture is indeed true.

## Conclusion

The present study investigated associations between three *PPP1R1B* polymorphisms and working memory ability measured by the WCST in a sample of Chinese students to demonstrate possible biogenetic mechanisms affecting working memory. We found that the rs12601930 genotypes were associated with five dimensions of the six studied WCST dimensions (except PR) and rs3764352 genotypes were associated with NPE and CLR dimensions in both the overall sample and males only, which indicate that participants with the TT genotype of the rs12601930 polymorphism and/or the GG genotype of the rs3764352 polymorphism have better ability of working memory compared to those with other genotypes. These results provided evidence that genetic variants in the DARPP-32 system could influence working memory by regulating the DA system and that this effect was affected by sex. Moreover, these results provide evidence for working memory as a promising candidate for the classification of cross-disease diagnosis of psychiatric diseases. However, this conclusion should be considered with caution due to the limited size and age range of the sample, the lack of participants with a psychiatric condition, the limited number of studied SNPs, and the use of neurocognitive assessment. Further studies of these three *PPP1R1B* SNPs and other SNPs associated with the DA system in a larger Chinese sample and other ethnic populations are needed to verify our findings and develop a more comprehensive understanding of the effects of *PPP1R1B* variants on working memory.

## Data availability statement

The raw data supporting the conclusions of this article are available upon request.

## Ethics statement

The studies involving human participants were reviewed and approved by the Ethics Committee of China Medical University. The participants provided their written informed consent to participate in this study.

## Author contributions

HM and MW: conceptualization and writing—review and editing. HM and WZ: methodology. HM: software, formal analysis, and visualization. XC and LZ: validation. HM, LZ, and MW: investigation. MW: resources, supervision, project administration, and funding acquisition. HM, RQ, WZ, and XC: data curation. HM and RQ: writing—original draft preparation. All authors contributed to the article and approved the submitted version.

## Footnotes

1
http://www.ncbi.nlm.nih.gov

## References

[B1] Ando J. Ono Y. Wright M. J. (2001). Genetic structure of spatial and verbal working memory. *Behav Genet*. 31 615–624. 10.1023/a:1013353613591 11838538

[B2] Arts B. Jabben N. Krabbendam L. van Os J. (2008). Meta-analyses of cognitive functioning in euthymic bipolar patients and their first-degree relatives. *Psychol. Med*. 38 771–785. 10.1017/S0033291707001675 17922938

[B3] Axelrod B. N. (2002). Are normative data from the 64-card version of the WCST comparable to the full WCST? *Clin. Neuropsychol*. 16 7–11. 10.1076/clin.16.1.7.8331 11992222

[B4] Baddeley A. (2012). Working memory: Theories, models, and controversies. *Annu. Rev. Psychol*. 63 1–29. 10.1146/annurev-psych-120710-100422 21961947

[B5] Beaulieu J. M. Sotnikova T. D. Marion S. Lefkowitz R. J. Gainetdinov R. R. Caron M. G. (2005). An Akt/beta-arrestin 2/PP2A signaling complex mediates dopaminergic neurotransmission and behavior. *Cell* 122 261–273. 10.1016/j.cell.2005.05.012 16051150

[B6] Berg E. A. (1948). A simple objective technique for measuring flexibility in thinking. *J. Gen. Psychol*. 39 15–22. 10.1080/00221309.1948.9918159 18889466

[B7] Bertolino A. Fazio L. Caforio G. Blasi G. Rampino A. Romano R. (2009). Functional variants of the dopamine receptor D2 gene modulate prefronto-striatal phenotypes in schizophrenia. *Brain* 132 417–425. 10.1093/brain/awn248 18829695 PMC2640212

[B8] Bolton A. D. Constantine-Paton M. (2018). Synaptic Effects of Dopamine Breakdown and Their Relation to Schizophrenia-Linked Working Memory Deficits. *Front. Synap. Neurosci*. 10:16. 10.3389/fnsyn.2018.00016 29950984 PMC6008544

[B9] Brainstorm C. Anttila V. Bulik-Sullivan B. Finucane H. K. Walters R. K. Bras J. (2018). Analysis of shared heritability in common disorders of the brain. *Science* 360:eaa8757. 10.1126/science.aap8757 29930110 PMC6097237

[B10] Brydges C. R. Ozolnieks K. L. Roberts G. (2021). Working Memory and Intraindividual Variability in Response Time Mediate Fluid Intelligence Deficits Associated With ADHD Symptomology. *J. Atten. Disord*. 25 63–72. 10.1177/1087054718772143 29708000

[B11] Cross-Disorder Group of the Psychiatric Genomics Consortoium (2013). Identification of risk loci with shared effects on five major psychiatric disorders: A genome-wide analysis. *Lancet* 381 1371–1379. 10.1016/S0140-6736(12)62129-1 23453885 PMC3714010

[B12] Cross-Disorder Group of the Psychiatric Genomics Consortoium S. H. Ripke S. Neale B. M. Faraone S. V. Purcell S. M. (2013). Genetic relationship between five psychiatric disorders estimated from genome-wide SNPs. *Nat. Genet*. 45 984–994. 10.1038/ng.2711 23933821 PMC3800159

[B13] Caldu X. Vendrell P. Bartres-Faz D. Clemente I. Bargallo N. Jurado M. A. (2007). Impact of the COMT Val108/158 Met and DAT genotypes on prefrontal function in healthy subjects. *Neuroimage* 37 1437–1444. 10.1016/j.neuroimage.2007.06.021 17689985

[B14] Cristofori I. Cohen-Zimerman S. Grafman J. (2019). Executive functions. *Handb. Clin. Neurol*. 163 197–219. 10.1016/B978-0-12-804281-6.00011-2 31590731

[B15] Curcic-Blake B. Swart M. Ter Horst G. J. Langers D. R. Kema I. P. Aleman A. (2012). Variation of the gene coding for DARPP-32 (PPP1R1B) and brain connectivity during associative emotional learning. *Neuroimage* 59 1540–1550. 10.1016/j.neuroimage.2011.08.036 21878394

[B16] Demakis G. J. (2003). A meta-analytic review of the sensitivity of the Wisconsin Card Sorting Test to frontal and lateralized frontal brain damage. *Neuropsychology* 17 255–264. 10.1037/0894-4105.17.2.255 12803431

[B17] Dumontheil I. Jensen S. K. Wood N. W. Meyer M. L. Lieberman M. D. Blakemore S. J. (2014). Preliminary investigation of the influence of dopamine regulating genes on social working memory. *Soc. Neurosci*. 9 437–451. 10.1080/17470919.2014.925503 24889756 PMC4131246

[B18] Dumontheil I. Kilford E. J. Blakemore S. J. (2020). Development of dopaminergic genetic associations with visuospatial, verbal and social working memory. *Dev. Sci*. 23:e12889. 10.1111/desc.12889 31336006 PMC7064996

[B19] Duncan J. Seitz R. J. Kolodny J. Bor D. Herzog H. Ahmed A. (2000). A neural basis for general intelligence. *Science* 289 457–460. 10.1126/science.289.5478.457 10903207

[B20] Emamian E. S. Hall D. Birnbaum M. J. Karayiorgou M. Gogos J. A. (2004). Convergent evidence for impaired AKT1-GSK3beta signaling in schizophrenia. *Nat. Genet*. 36 131–137. 10.1038/ng1296 14745448

[B21] Furman D. J. Zhang Z. Chatham C. H. Good M. Badre D. Hsu M. (2021). Augmenting Frontal Dopamine Tone Enhances Maintenance over Gating Processes in Working Memory. *J. Cogn. Neurosci*. 33 1753–1765. 10.1162/jocn_a_01641 33054556 PMC8647935

[B22] Giovannetti E. Zucali P. A. Peters G. J. Cortesi F. D’Incecco A. Smit E. F. (2010). Association of polymorphisms in AKT1 and EGFR with clinical outcome and toxicity in non-small cell lung cancer patients treated with gefitinib. *Mol. Cancer Ther*. 9 581–593. 10.1158/1535-7163.MCT-09-0665 20159991

[B23] Girault J. A. Nairn A. C. (2021). DARPP-32 40 years later. *Adv. Pharmacol*. 90 67–87. 10.1016/bs.apha.2020.09.004 33706939

[B24] Gold J. M. Carpenter C. Randolph C. Goldberg T. E. Weinberger D. R. (1997). Auditory working memory and Wisconsin Card Sorting Test performance in schizophrenia. *Arch. Gen. Psych*. 54 159–165. 10.1001/archpsyc.1997.01830140071013 9040284

[B25] Grant D. A. Berg E. A. (1948). A behavioral analysis of degree of reinforcement and ease of shifting to new responses in a Weigl-type card-sorting problem. *J. Exp. Psychol*. 38 404–411. 10.1037/h0059831 18874598

[B26] Gray J. R. Chabris C. F. Braver T. S. (2003). Neural mechanisms of general fluid intelligence. *Nat. Neurosci*. 6 316–322. 10.1038/nn1014 12592404

[B27] Greengard P. Allen P. B. Nairn A. C. (1999). Beyond the dopamine receptor: The DARPP-32/protein phosphatase-1 cascade. *Neuron* 23 435–447. 10.1016/s0896-6273(00)80798-9 10433257

[B28] Gur R. E. Calkins M. E. Gur R. C. Horan W. P. Nuechterlein K. H. Seidman L. J. (2007). The Consortium on the Genetics of Schizophrenia: Neurocognitive endophenotypes. *Schizophr. Bull*. 33 49–68. 10.1093/schbul/sbl055 17101692 PMC2632287

[B29] Hammerschlag A. R. de Leeuw C. A. Middeldorp C. M. Polderman T. J. C. (2020). Synaptic and brain-expressed gene sets relate to the shared genetic risk across five psychiatric disorders. *Psychol. Med*. 50 1695–1705. 10.1017/S0033291719001776 31328717 PMC7408577

[B30] Harris S. L. Gil G. Robins H. Hu W. Hirshfield K. Bond E. (2005). Detection of functional single-nucleotide polymorphisms that affect apoptosis. *Proc. Natl. Acad. Sci. U.S.A*. 102 16297–16302. 10.1073/pnas.0508390102 16260726 PMC1283473

[B31] Hernandez-Lopez S. Tkatch T. Perez-Garci E. Galarraga E. Bargas J. Hamm H. (2000). D2 dopamine receptors in striatal medium spiny neurons reduce L-type Ca2+ currents and excitability via a novel PLC[beta]1-IP3-calcineurin-signaling cascade. *J. Neurosci*. 20 8987–8995. 11124974 10.1523/JNEUROSCI.20-24-08987.2000 PMC6773013

[B32] Houlihan L. M. Harris S. E. Luciano M. Gow A. J. Starr J. M. Visscher P. M. (2009). Replication study of candidate genes for cognitive abilities: The Lothian Birth Cohort 1936. *Genes Brain Behav*. 8 238–247. 10.1111/j.1601-183X.2008.00470.x 19077115

[B33] Huang Y. Li J. Ma H. Zhao X. Wang Y. Jin Q. (2013). Association between PPP1R1B polymorphisms and defense mechanisms in healthy Chinese-Han subjects. *J. Mol. Neurosci*. 49 618–624. 10.1007/s12031-012-9907-1 23080070

[B34] Ishikawa M. Mizukami K. Iwakiri M. Asada T. (2007). Immunohistochemical and immunoblot analysis of Dopamine and cyclic AMP-regulated phosphoprotein, relative molecular mass 32,000 (DARPP-32) in the prefrontal cortex of subjects with schizophrenia and bipolar disorder. *Prog. Neuropsychopharmacol. Biol. Psych*. 31 1177–1181. 10.1016/j.pnpbp.2007.04.013 17521792

[B35] Jolly A. E. Scott G. T. Sharp D. J. Hampshire A. H. (2020). Distinct patterns of structural damage underlie working memory and reasoning deficits after traumatic brain injury. *Brain* 143 1158–1176. 10.1093/brain/awaa067 32243506 PMC7174032

[B36] Karlsgodt K. H. Bachman P. Winkler A. M. Bearden C. E. Glahn D. C. (2011). Genetic influence on the working memory circuitry: Behavior, structure, function and extensions to illness. *Behav. Brain Res*. 225 610–622. 10.1016/j.bbr.2011.08.016 21878355 PMC3545474

[B37] Kimberg D. Y. D’Esposito M. (2003). Cognitive effects of the dopamine receptor agonist pergolide. *Neuropsychologia* 41 1020–1027. 10.1016/s0028-3932(02)00317-2 12667537

[B38] Klaus K. Pennington K. (2019). Dopamine and Working Memory: Genetic Variation, Stress and Implications for Mental Health. *Curr. Top. Behav. Neurosci*. 41 369–391. 10.1007/7854_2019_113 31502081

[B39] Kopp B. Lange F. Steinke A. (2021). The Reliability of the Wisconsin Card Sorting Test in Clinical Practice. *Assessment* 28 248–263. 10.1177/1073191119866257 31375035 PMC7780274

[B40] Kunii Y. Hino M. Matsumoto J. Nagaoka A. Nawa H. Kakita A. (2019). Differential protein expression of DARPP-32 versus Calcineurin in the prefrontal cortex and nucleus accumbens in schizophrenia and bipolar disorder. *Sci. Rep*. 9:14877. 10.1038/s41598-019-51456-7 31619735 PMC6796065

[B41] Kunii Y. Hyde T. M. Ye T. Li C. Kolachana B. Dickinson D. (2014). Revisiting DARPP-32 in postmortem human brain: Changes in schizophrenia and bipolar disorder and genetic associations with t-DARPP-32 expression. *Mol. Psych*. 19 192–199. 10.1038/mp.2012.174 23295814

[B42] Lange F. Kroger B. Steinke A. Seer C. Dengler R. Kopp B. (2016). Decomposing card-sorting performance: Effects of working memory load and age-related changes. *Neuropsychology* 30 579–590. 10.1037/neu0000271 26866348

[B43] Li J. Chen C. Chen C. He Q. Li H. Li J. (2011a). Neurotensin receptor 1 gene (NTSR1) polymorphism is associated with working memory. *PLoS One* 6:e17365. 10.1371/journal.pone.0017365 21394204 PMC3048867

[B44] Li J. Ma H. Zhou H. Huang Y. Wu L. Li J. (2011b). Association between DARPP-32 gene polymorphism and personality traits in healthy Chinese-Han subjects. *J. Mol. Neurosci*. 44 48–52. 10.1007/s12031-011-9505-7 21369787

[B45] Li Y. Yang N. Zhang Y. Xu W. Cai L. (2021). The Relationship Among Trait Mindfulness, Attention, and Working Memory in Junior School Students Under Different Stressful Situations. *Front. Psychol*. 12:558690. 10.3389/fpsyg.2021.558690 33737892 PMC7960675

[B46] Lin R. Learman L. N. Na C. H. Renuse S. Chen K. T. Chen P. Y. (2021). Persistently Elevated mTOR Complex 1-S6 Kinase 1 Disrupts DARPP-32-Dependent D1 Dopamine Receptor Signaling and Behaviors. *Biol. Psych*. 89 1058–1072. 10.1016/j.biopsych.2020.10.012 33353667 PMC8076344

[B47] Liozidou A. Potagas C. Papageorgiou S. G. Zalonis I. (2012). The role of working memory and information processing speed on wisconsin card sorting test performance in Parkinson disease without dementia. *J. Geriatr. Psych. Neurol*. 25 215–221. 10.1177/0891988712466456 23220907

[B48] Ma H. Huang Y. Li X. Lin A. Cong Z. Zhu G. (2015). A case-control study of the association between DARPP-32 gene polymorphisms and alcohol dependence in Chinese Han subjects. *Psych. Res*. 229 1052–1054. 10.1016/j.psychres.2015.08.026 26304024

[B49] Ma H. Li X. Lin A. Yuan Z. Zhou J. Yang X. (2017). Associations Between PPP1R1B Gene Polymorphisms and Anxiety Levels in the Chinese Population. *Neurosci. Bull*. 33 107–110. 10.1007/s12264-016-0088-8 27995567 PMC5567550

[B50] Matsuo K. Glahn D. C. Peluso M. A. Hatch J. P. Monkul E. S. Najt P. (2007). Prefrontal hyperactivation during working memory task in untreated individuals with major depressive disorder. *Mol. Psych*. 12 158–166. 10.1038/sj.mp.4001894 16983390

[B51] Meyer-Lindenberg A. Straub R. E. Lipska B. K. Verchinski B. A. Goldberg T. Callicott J. H. (2007). Genetic evidence implicating DARPP-32 in human frontostriatal structure, function, and cognition. *J. Clin. Invest*. 117 672–682. 10.1172/JCI30413 17290303 PMC1784004

[B52] Ouimet C. C. LaMantia A. S. Goldman-Rakic P. Rakic P. Greengard P. (1992). Immunocytochemical localization of DARPP-32, a dopamine and cyclic-AMP-regulated phosphoprotein, in the primate brain. *J. Comp. Neurol*. 323 209–218. 10.1002/cne.903230206 1328330

[B53] Ouimet C. C. Langley-Gullion K. C. Greengard P. (1998). Quantitative immunocytochemistry of DARPP-32-expressing neurons in the rat caudatoputamen. *Brain Res*. 808 8–12. 10.1016/s0006-8993(98)00724-0 9795103

[B54] Ouimet C. C. Miller P. E. Hemmings H. C. Jr. Walaas S. I. Greengard P. (1984). DARPP-32, a dopamine- and adenosine 3’:5’-monophosphate-regulated phosphoprotein enriched in dopamine-innervated brain regions. III. Immunocytochemical localization. *J. Neurosci.* 4 111–124. 10.1523/JNEUROSCI.04-01-00111.1984 6319625 PMC6564746

[B55] Rabiee A. Vasaghi-Gharamaleki B. Samadi S. A. Amiri-Shavaki Y. Alaghband-Rad J. (2020). Working Memory Deficits and its Relationship to Autism Spectrum Disorders. *Iran J. Med. Sci*. 45 100–109. 10.30476/IJMS.2019.45315 32210486 PMC7071553

[B56] Rabin L. A. Barr W. B. Burton L. A. (2005). Assessment practices of clinical neuropsychologists in the United States and Canada: A survey of INS, NAN, and APA Division 40 members. *Arch. Clin. Neuropsychol*. 20 33–65. 10.1016/j.acn.2004.02.005 15620813

[B57] Ramos A. A. Hamdan A. C. Machado L. (2020). A meta-analysis on verbal working memory in children and adolescents with ADHD. *Clin. Neuropsychol*. 34 873–898. 10.1080/13854046.2019.1604998 31007130

[B58] Ricker T. J. Nieuwenstein M. R. Bayliss D. M. Barrouillet P. (2018). Working memory consolidation: Insights from studies on attention and working memory. *Ann. N Y Acad. Sci*. 1424 8–18. 10.1111/nyas.13633 29635694

[B59] Ruiz-Sanchez E. Jimenez-Genchi J. Alcantara-Flores Y. M. Castaneda-Gonzalez C. J. vina-Cervantes C. L. A. Yescas P. (2021). Working memory deficits in schizophrenia are associated with the rs34884856 variant and expression levels of the NR4A2 gene in a sample Mexican population: A case control study. *BMC Psych*. 21:86. 10.1186/s12888-021-03081-w 33563249 PMC7871565

[B60] Saykin A. J. Shtasel D. L. Gur R. E. Kester D. B. Mozley L. H. Stafiniak P. (1994). Neuropsychological deficits in neuroleptic naive patients with first-episode schizophrenia. *Arch. Gen. Psych*. 51 124–131. 10.1001/archpsyc.1994.03950020048005 7905258

[B61] Schork A. J. Won H. Appadurai V. Nudel R. Gandal M. Delaneau O. (2019). A genome-wide association study of shared risk across psychiatric disorders implicates gene regulation during fetal neurodevelopment. *Nat. Neurosci*. 22 353–361. 10.1038/s41593-018-0320-0 30692689 PMC6497521

[B62] Schwarz E. Tost H. Meyer-Lindenberg A. (2016). Working memory genetics in schizophrenia and related disorders: An RDoC perspective. *Am. J. Med. Genet B Neuropsychiatr. Genet*. 171B 121–131. 10.1002/ajmg.b.32353 26365198

[B63] Soderqvist S. Bergman Nutley S. Peyrard-Janvid M. Matsson H. Humphreys K. Kere J. (2012). Dopamine, working memory, and training induced plasticity: Implications for developmental research. *Dev. Psychol*. 48 836–843. 10.1037/a0026179 22103304

[B64] Sogut M. Goksun T. Altan-Atalay A. (2021). The role of numeracy skills on the Wisconsin card sorting test (WCST) performances of 5- to 8-Year-old turkish children. *Br. J. Dev. Psychol*. 39 231–246. 10.1111/bjdp.12353 33058246

[B65] Svenningsson P. Nairn A. C. Greengard P. (2005). DARPP-32 mediates the actions of multiple drugs of abuse. *AAPS J*. 7 E353–E360. 10.1208/aapsj070235 16353915 PMC2750972

[B66] Svenningsson P. Nishi A. Fisone G. Girault J. A. Nairn A. C. Greengard P. (2004). DARPP-32: An integrator of neurotransmission. *Annu. Rev. Pharmacol. Toxicol*. 44 269–296. 10.1146/annurev.pharmtox.44.101802.121415 14744247

[B67] Tan H. Y. Nicodemus K. K. Chen Q. Li Z. Brooke J. K. Honea R. (2008). Genetic variation in AKT1 is linked to dopamine-associated prefrontal cortical structure and function in humans. *J. Clin. Invest*. 118 2200–2208. 10.1172/JCI34725 18497887 PMC2391279

[B68] Thurston-Snoha B. J. Lewine R. R. (2007). Intact Wisconsin Card Sorting Test performance: Implications for the role of executive function in schizophrenia. *Br. J. Clin. Psychol*. 46 361–369. 10.1348/014466507X173772 17535528

[B69] Torres K. C. Souza B. R. Miranda D. M. Nicolato R. Neves F. S. Barros A. G. (2009). The leukocytes expressing DARPP-32 are reduced in patients with schizophrenia and bipolar disorder. *Prog. Neuropsychopharmacol. Biol. Psych*. 33 214–219. 10.1016/j.pnpbp.2008.10.020 19059449

[B70] Vance A. Winther J. (2021). Spatial working memory performance in children and adolescents with major depressive disorder and dysthymic disorder. *J. Affect. Disord*. 278 470–476. 10.1016/j.jad.2020.09.093 33017674

[B71] Vaughan R. S. Laborde S. (2021). Attention, working-memory control, working-memory capacity, and sport performance: The moderating role of athletic expertise. *Eur. J. Sport Sci*. 21 240–249. 10.1080/17461391.2020.1739143 32129718

[B72] Verhagen J. Leseman P. (2016). How do verbal short-term memory and working memory relate to the acquisition of vocabulary and grammar? A comparison between first and second language learners. *J. Exp. Child. Psychol*. 141 65–82. 10.1016/j.jecp.2015.06.015 26340756

[B73] Vijayraghavan S. Wang M. Birnbaum S. G. Williams G. V. Arnsten A. F. (2007). Inverted-U dopamine D1 receptor actions on prefrontal neurons engaged in working memory. *Nat. Neurosci*. 10 376–384. 10.1038/nn1846 17277774

[B74] Walaas S. I. Aswad D. W. Greengard P. (1983). A dopamine- and cyclic AMP-regulated phosphoprotein enriched in dopamine-innervated brain regions. *Nature* 301 69–71. 10.1038/301069a0 6296685

[B75] Wilkosc M. Hauser J. Tomaszewska M. Dmitrzak-Weglarz M. Skibinska M. Szczepankiewicz A. (2010). Influence of dopaminergic and serotoninergic genes on working memory in healthy subjects. *Acta Neurobiol. Exp.* 70 86–94. 10.55782/ane-2010-1777 20407490

[B76] Wu S. Upadhyay N. Lu J. Jiang X. Li S. Qing Z. (2020). Interaction of Catechol-O-methyltransferase Val(158) Met polymorphism and sex influences association of parietal intrinsic functional connectivity and immediate verbal memory. *Brain Behav*. 10:e01784. 10.1002/brb3.1784 32772512 PMC7559624

[B77] Youngs M. A. Lee S. E. Mireku M. O. Sharma D. Kramer R. S. S. (2021). Mindfulness Meditation Improves Visual Short-Term Memory. *Psychol. Rep*. 124 1673–1686. 10.1177/0033294120926670 32448056 PMC8242403

[B78] Zakic Milas D. Milas G. (2019). Working Memory in Patients with Schizophrenia and Bipolar Affective Disorder: Quantitative or Qualitative Differences? *Psychiatr. Danub*. 31 54–61. 10.24869/psyd.2019.54 30948690

[B79] Zhang Y. Bertolino A. Fazio L. Blasi G. Rampino A. Romano R. (2007). Polymorphisms in human dopamine D2 receptor gene affect gene expression, splicing, and neuronal activity during working memory. *Proc. Natl. Acad. Sci. U.S.A*. 104 20552–20557. 10.1073/pnas.0707106104 18077373 PMC2154469

